# Online Anomaly Detection System for Mobile Networks

**DOI:** 10.3390/s20247232

**Published:** 2020-12-17

**Authors:** Jesús Burgueño, Isabel de-la-Bandera, Jessica Mendoza, David Palacios, Cesar Morillas, Raquel Barco

**Affiliations:** 1 Department of Communications Engineering, University of Malaga, 29071 Málaga, Spain; ibanderac@ic.uma.es (I.d.-l.-B.); jmr@ic.uma.es (J.M.); rbm@ic.uma.es (R.B.); 2 Tupl Spain S.L., Tupl Inc., 29010 Málaga, Spain; david.palacios@tupl.com (D.P.); cesar.morillas@tupl.com (C.M.)

**Keywords:** anomaly detection, network operation, LTE, self-healing

## Abstract

The arrival of the fifth generation (5G) standard has further accelerated the need for operators to improve the network capacity. With this purpose, mobile network topologies with smaller cells are currently being deployed to increase the frequency reuse. In this way, the number of nodes that collect performance data is being further risen, so the number of metrics to be managed and analyzed is being highly increased. Therefore, it is fundamental to have tools that automatically inform the network operator of the relevant information within the vast amount of metrics collected. The continuous monitoring of the performance indicators and the automatic detection of anomalies is especially important for network operators to prevent the network degradation and user complaints. Therefore, this paper proposes a methodology to detect and track anomalies in the mobile networks performance indicators online, i.e., in real time. The feasibility of this system was evaluated with several performance metrics and a real LTE Advanced dataset. In addition, it was also compared with the performances of other state-of-the-art anomaly detection systems.

## 1. Introduction

In recent years, the number of traditional users which are connected to the mobile networks has been constantly increasing. In addition, mobile networks are experiencing a massive increase in automatic devices from many different areas, such as sensor networks, wearables and connected vehicles [1]. This trend is referred to as Internet of Things (IoT). In this way, IoT and the arrival of 5G have driven demand for a wider range of services. To address these challenges, communications with higher data rates, more available bandwidth and lower interference have to be established. These challenges lead to the need for improving the network capacity by deployment of mobile networks with lower inter-site distances in order to increase the frequency reuse [2]. This new trend is cited as network densification and it will enable the fulfillment of more demanding user requirements over the next few years. The network densification will involve the deployment of many more base stations with lower inter-site distances. Thus, this will allow better resource reuse and an improvement in network capacity. Likewise, each base station will provide resources to a lower number of users, improving the available bandwidth per user.

Since the number of nodes that collect data on the network performance will be highly increased, the amount of metrics to be managed and analyzed will be further risen. In this sense, each node collects hundreds of the most important network performance indicators, known as key performance indicators (KPIs). Hence, it is essential to have tools that automatically bring out the relevant information that is hidden among the vast amount of collected metrics [3]. With this objective, different approaches can be addressed. In a centralized approach, data collected in each network site are delivered to the upper network equipment, where data are analyzed by the developed tool. This approach implies a data overloading in this central equipment but also enables one to use more complex tools that correlate data from different sites. On the other hand, a distributed approach allows one to minimize the delay of results since the developed tool is deployed in each network’s area. In this case, if the developed tool has to be integrated into existing nodes so as not to shoot up the operating expenses (OPEX) of the network, it should require low computational cost.

In addition, data analysis is more important than ever, as emerging functionalities are being implemented in mobile networks, such as software defined networking (SDN) and network function virtualization (NFV) [4], which allow that the available network resources and services may be modified in real time. Thus, this causes an instantaneous effect in collected KPIs. Similarly, a change in network configuration made by the operator, and external influences such as social events, can also lead to significant changes in network performance. Hence, a tool that automatically detects behavioral changes in KPIs before users complain about the degraded quality of experience is essential [5]. This tool should trigger an alert when the current data show an unusual change of behavior in any KPI. In this way, network experts could make decisions focused on correcting this anomalous effect if necessary. This implies that the detection of the anomaly must be done in real time; that is, the start of an anomaly must be indicated to the operator as soon as the first anomalous samples are detected in a KPI. However, most anomaly detection studies focus on the identification of anomalies once the complete time series of the KPI is available. On the other hand, the subsequent tracking of the KPI could notify the network operator of its status in the future, allowing it to know when the anomaly has ended.

To automatically identify anomalous samples, one of the best options is to use historical data labeled by network experts. These datasets classify the samples of a KPI into normal and anomalous, so that the relationship between the samples of the KPI and the label can be known to automatically classify new inputs [6]. However, historical data are usually not labeled, excluding those cases of interest that have been studied and analyzed in depth by the operator. Therefore, a large proportion of published studies add synthetic anomalies to obtain datasets with classified samples [7,8]. Nonetheless, these fictitious anomalies do not faithfully coincide with the real cases. On the other hand, other studies propose methods of anomaly detection that do not use labeled datasets [9,10,11]. Hence, the opinions of the network experts are not considered to distinguish the anomalies in these cases. However, the main drawbacks of these techniques are that they mainly detect the most noticeable outliers of the KPIs and that several anomalies identified by the network experts are omitted by these methods [8,9,11]. In the opinion of the network experts, level shift anomalies, which are characterized by some temporary increase or decrease in the KPI mean over the duration of the anomaly, and anomalies maintained over time when the KPIs totally change their trends and not just their means, are much more damaging than occasional outliers because the behavior of the network changes over a period of time (they are not one-off anomalies). Therefore, anomalies maintained over time have more impact on the quality of the experience perceived by users, as the new behavior may last until the network operator realizes the reason for such a change and makes a decision about it. Thus, the fastest possible detection of an anomaly is very critical in mobile networks, as mentioned above.

In addition to anomalies, network behavior patterns may change over time depending on the month of the year, due to changes in the scenario covered by a cell, etc. Hence, it is mandatory that the deployed technique is updated over time without any interruption to continue operating in a feasible manner. This is a major constraint for some techniques that must be retrained with new labeled data or for other approaches that must be manually tuned [12]. This implies a significant showstopper for these techniques to be deployed through a mobile network with a large number of nodes. This is even more challenging for high-cost computing techniques [13]. To reduce computational costs, some approaches decide whether a cell is anomalous or not based on a combination of a reduced set of KPIs [7,14]. However, they do not differentiate between KPIs that function normally and those that do not.

Given these limitations, this paper proposes a novel methodology to identify anomalies in KPIs of mobile networks in real time. The proposed solution monitors the state of an anomaly from its detection to its end. The system consists of two stages: a learning block of the seasonal patterns of the KPIs and a block that detects and tracks anomalies as each new sample of the KPIs is collected. Additionally, the latter block includes a mechanism to automatically adapt the functioning of the whole system to new network behaviors in run time. This allows one to work feasibly without manual intervention in the long term. This study makes the following contributions:

- The proposed methodology combines different mechanisms with the objective of identifying the beginnings of the most harmful anomalies as each new KPI sample is collected, and then monitoring their states until they end. In this sense, the detection of anomalies maintained over time has been prioritized over chance outliers, according to the requirements of mobile network operators. Furthermore, the proposed methodology aims at minimizing the number of anomalies that the system overlooks while maintaining a low false positive rate, so that network operators only expend resources dealing with true anomalies. To evaluate the performance of the proposed system, the measures most often cited in the literature on machine learning have been used. It has been evaluated with an actual LTE Advanced dataset, wherein real anomalies have been labeled by network experts and no synthetic anomalies have been manually injected. In addition, the performance of the proposed system was compared with the performances of state-of-the-art approaches. The results demonstrate that the proposed methodology identifies about 25% more total anomalies in mobile networks than the other approaches, while maintaining a high level of precision and a low number of false positives.
- The proposed system has been designed to be easily implemented with a low computational cost in the current network nodes already deployed. Likewise, the system is scalable and can be used for a different number of indicators, since the system detects anomalies for each KPI without taking into account the rest of the KPIs. In addition, it automatically adapts to new network behavior profiles without any manual intervention or feedback. Given these properties, the system can also be adapted to other use cases, such as IoT or device-to-device decentralized networks. Likewise, its use can be extended to next generation networks.

The rest of the paper is organized as follows. Section 2 analyzes the related works in the field. In Section 3 an introduction about anomaly detection in real time is presented. Section 4 details the proposed system. Its subsections describe each of the parts that form the whole system. In Section 5 the methodology used to evaluate the system is defined. This section presents the dataset and the metrics used to analyze the performance in addition to the rest of systems that were tested as well, whereas Section 6 analyzes the results. Section 7 concludes with a summary and directions for future work.

## 2. Related Work

There is abundant literature in the field of anomaly detection. However, most studies focus on proposing solutions to identify anomalies once the method has the complete time series of an indicator; i.e., they detect an anomaly once its start and end samples have been collected. On the contrary, fewer studies focus on the detection of anomalies in real time, that is, on the triggering of an alert as soon as the anomalous samples are collected. In this section, these last studies related to the proposed method are analyzed.

One of the fundamental aspects to developing anomaly detection systems is to have labeled datasets, either to train a method or to test its performance. Due to the lack of anomalies labeled by network experts, most studies add synthetic anomalies in one of these two stages. In this sense, the authors of [7] proposed a system trained with synthetic anomalies, based on regression analysis. Multiple correlated KPIs are needed to detect anomalies, so this technique cannot be applied to different KPIs separately. In a similar manner, the authors of [14] introduced a centralized framework that uses self-organizing maps (SOM) and the k-medoids technique. It uses a set of KPIs from each cell as input. Hence, it detects the cells that have anomalous behavior, but it also does not detect anomalies at the KPI level.

On the other hand, ref. [8] only used a dataset with synthetic anomalies to evaluate the system. In this case, an autoregressive integrated moving average (ARIMA) algorithm is used to forecast the expected value of the next coming sample. Then, the system makes a decision based on the similarity between the forecast sample and the next real sample. The results show that it achieves high precision for chance outliers but low precision for level shift anomalies, which are some of the most damaging anomalies in mobile networks, as introduced in the previous section. In a similar way, only occasional outliers are detected by the methodology proposed in [9]. In this case, a dynamic k-nearest neighbors (k-NN) algorithm is used.

Based on different algorithms but achieving poor performance as well, the authors of [11] proposed two anomaly detection techniques. They estimate the temporal properties of an input data stream through adaptive learning. Nevertheless, each method uses a different way of calculating its sliding window. They were tested with Internet traffic datasets, and the results show that although the performances of both methods are good for non-periodic streams, they are especially poor for periodic streams because of the high false positive rates. Therefore, they are not suitable for mobile networks, wherein a large number of periodic KPIs are usually collected. In addition, the results are even worse when there are level shift anomalies in periodic streams. On the other hand, the false positive rate is also too high (more than 14%) in [15]. In this case, the system is focused on identifying unusually low or high user traffic areas in mobile networks. Nevertheless, the high false positive rate implies that the system often warns the network operator of non-existent troubles, shooting up network OPEX.

In a completely different way, the authors of [10] introduced a system that includes a set of fictitious degradation patterns. These synthetic patterns are then compared with a KPI. If they are sufficiently correlated, the KPI is identified as degraded. In this case, the performance of the method is highly limited by the patterns defined for each KPI by network experts. These experts also have an important role in the generic system proposed in [12]. This system detects anomalies in real time, but its configuration must be constantly tuned using the feedback provided by experts in order to reach a good performance over time, increasing network OPEX. On the other hand, the authors of [13] introduced a system based on deep neural networks to detect outages and congested cells. This approach is computationally costly to deploy on current equipment at each network site due to the large amount of operations to be processed. In addition, a huge amount of new labeled data is required each time the network behavior profile changes.

Alternatively, many authors have proposed their own anomaly detection system and have uploaded their corresponding code to online repositories so that any user can test them with different datasets. Thus, these approaches can be accurately compared with the methodology proposed in this paper. In this manner, a Python package for anomaly detection is shared in [16]. This technique focuses on identifying new, different behavioral patterns by using singular spectrum transformation (SST). It mainly detects anomalies when the frequency of the analyzed indicator changes considerably. Another Python package is shared in [17]. In this case, the authors proposed a methodology that combines different detection algorithms to create a multi-purpose system that detects any type of anomaly. Therefore, the decision will be made based on the criteria of different algorithms, which implies that many anomalies are detected by one algorithm but not confirmed at subsequent stages. Thus, the number of true anomalies detected decreases in exchange for a decrease in the number of false anomalies confirmed. In addition to Python packages, the authors of [18] presented an R package to detect anomalies by using seasonal hybrid extreme studentized deviate (S-H-ESD). This technique calculates the statistical values that are used to decide whether a sample is anomalous based on the percentage of anomalous samples that the analyzed indicator usually has. Therefore, it must be configured with the expected percentage of anomalies in the KPI. This implies that the number of anomalies indicated by the proposed method is close to the set value, resulting in poor performance when the number of actual anomalies increases noticeably or approaches zero.

Given the limitations of the related work, the need to develop an online anomaly detection system that can already be deployed on real mobile networks with low computing cost impact is confirmed. Especially, this system should outperform the state-of-the-art approaches in terms of detection of the most harmful anomalies, according to the requirements of mobile network operators. In the same way, the system should automatically adapt to new network behavior profiles without any manual intervention or feedback.

## 3. Online Anomaly Detection

An anomaly is defined as an atypical behavior that is significantly different from the previous normal behavior of a KPI over a period of time. Hence, it is mandatory to study the previous KPI behavior to decide whether the current one is anomalous or not. An anomaly may imply a negative change in the network, such as a decrease in the cell availability. Conversely, an anomaly may also cause a positive effect, like an unusually low interference level. However, this positive fact may have been caused by a negative event in another cell, e.g., the outage of a neighboring cell. Therefore, all anomalies should be communicated to the network operator.

Regarding the study of the previous behavior of the KPIs, seasonal KPIs are very common in mobile networks. The periodic behavior of these KPIs reflects the typical users’ behavior and the management policies of the network operator. Additionally, these KPIs are typically business-related, e.g., the number of connected devices or throughput per user. On the other hand, the seasonal patterns may change or shift over time. For example, the number of connected devices may rely on the month of the year; e.g., the number of users for the same cell may be different in August than in March. Thus, it is critical that the KPI patterns are characterized and updated over time. In this sense, the authors of [19] proposed a system which detects the different seasonal patterns of a KPI in real time. This system uses a density-based spatial clustering of applications with noise (DBSCAN) algorithm. Moreover, if the traffic profile changes, the system will automatically adapt to new seasonal patterns. Besides, ref. [20] proposed a system that updates the normal behavior data when a system is reconfigured. An algorithm based on suffix trees was developed in this case.

Finally, the requirements of an ideal online anomaly detection system are shown below:

- **Detection of all anomalies as soon as possible**: If the anomaly is detected earlier, the network operator can take corrective action sooner. In this way, users will be affected for a shorter time.
- **False alerts must be minimized**: The network operator must minimize the wastage of resources on non-existent troubles.
- **Automatic adaptation to new behaviors**: If the KPI’s normal behavior changes over time, the system must automatically adapt to the new behavior.
- **No parameter tuning must be manually made at runtime**: Once the system is initialized, it should work reliably without manual assistance.

## 4. Proposed Methodology

**Figure 1:**
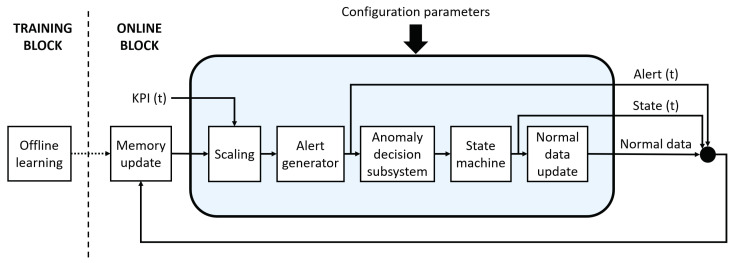
System diagram.

The proposed system aims at identifying the network anomalies and deciding the state of each KPI in real time. Thus, it will ideally be deployed in every node in order to monitor the KPIs of each cell. Figure 1 shows the block diagram of the proposed system, which is applicable to a single KPI. However, this can be replicated to monitor several KPIs. The system consists of a training block and an online block. The training block enables the initialization of the system. Once it is initialized, the online block can work without manual intervention over time. The online block consists of different subsystems. Firstly, the new KPI sample is scaled. If this sample is possibly anomalous, an alert will be triggered later by Alert generator. Anomaly decision subsystem then determines whether the new sample is definitely anomalous or not based on the previously generated alerts and the KPI state. Next, the KPI state is updated in State machine. Thus, the identification of anomalies will be tackled through the combined work of the last three subsystems mentioned. Finally, seasonal patterns are updated by Normal data update. The following subsections detail all the parts, and the inputs of the system.

### 4.1. Training Block

This subsection explains the process that must be done to initialize the system. To carry out this process, the following two values are required:

- **“T”**: Period of the main seasonal pattern of the KPI. It can be calculated with the system proposed in [19] that was introduced in the previous section.
- **“S”**: Number of previous KPI samples that the system will use to detect new anomalous behaviors. They must have been previously stored by the network operator. To ensure ideal system performance, most of this data should contain normal behavior. In the same way, the more previous KPI samples the operator can provide, the more reliable the detection of anomalies.

The training block aims at calculating and storing in memory each initial version of “avg_buffer” that will be used by the online block. Each “avg_buffer” is a buffer of “T” values based on each KPI period. Each of the values is calculated as a mean of the previous KPI samples corresponding to the same instant of time; e.g., if “T” is 24 because the KPI period is 24 h and the network operator collects one sample per hour, the first value of the “avg_buffer” will be the mean of the previous KPI samples corresponding to mid-point of the whole set of “S" samples. Hence, “S” samples will be used for calculating “T” average values. Consequently, as “S” increases, the average values will be calculated using more samples and the anomaly detection will be more reliable. However, the initial computational cost will grow.

### 4.2. Inputs of the Online Block

The online block takes three input sets. The first two sets include the new value of the KPI and the content of the memory, whereas the third set consists of the configuration parameters. The three sets are detailed as following:

- KPI (t): The new sample of the KPI.
- The content of the memory:
  – **“avg_buffer”**: Buffer composed of the “T” average values of the KPI for every instant of time over an entire period. It should be pointed out that the system must storage two buffers in the memory for different traffic patterns. One buffer will be used for weekdays and another for weekends and holidays.
  – **“d_buffer”**: Buffer composed of the last “T” values of “d”. This value “d” represents the difference between the new KPI sample and its corresponding sample in “avg_buffer”. It is further detailed in Alert generator.
  – **“alert_buffer”**: Buffer that stores the alerts triggered over time by Alert generator.
  – **“state_buffer”**: Buffer that stores the KPI state defined over time by State machine.
- The configuration parameters of the system:
  – **“k”**: Factor used in Scaling to increase or decrease the difference between the minimum and maximum values obtained after scaling. It is an integer value.
  – **“max_dif”**: Maximum difference that can exist between the new KPI sample and its corresponding average value to be considered as a normal sample.
  **“max_lag”**: Number of normal samples that must be received to leave an anomaly.
  – **“th_low”**: Threshold used in Alert generator to consider the triggering of a low alert.
  – **“th_med”**: Threshold used in Alert generator to consider the triggering of a medium alert.
  – **“th_high”**: Threshold used in Alert generator to consider the triggering of a high alert.

### 4.3. Online Block

Once the system is initialized, the online block is run every time a new KPI sample is received. Each of the parts that form the online block is detailed below.

#### 4.3.1. Memory Update

At first, the system memory must store the two “avg_buffer” values which are calculated when the system is initialized (the former for weekdays and the latter for weekends and holidays). Once the online block is running, Normal data update decides whether the corresponding “avg_buffer” value must be updated in each iteration. On the other hand, “d_buffer”, “alert_buffer” and “state_buffer” are updated in all iterations. In these cases, the new values are stored at the end of their corresponding buffer. In this way, Alert generator stores the new “d” value at “d_buffer”. It also stores at “alert_buffer” whether or not an alert has been triggered in this iteration. In case an alert has been generated, the type of this alert will be the stored value. Finally, State machine stores the current KPI state at “state_buffer”.

#### 4.3.2. Scaling

This stage is responsible for scaling the new KPI sample and “avg_buffer” in each iteration. Thus, the system works with scaled values although the memory stores the original ones.

At first, both “*min*” and “*max*” values are calculated as Equations (1) and (2) show. In these equations, $\bar{X}$ and $S_{x}$ indicate the mean and the standard deviation of the “S” values that are used to calculate “avg_buffer”. Once both values have been calculated, Equation (3) is used to scale the new KPI sample and “avg_buffer”. These scaled values will be used by the following stages.

(1) $$min=\bar{X}-k\cdot S_{x}$$

(2) $$max=\bar{X}+k\cdot S_{x}$$

(3) $$value^{\prime }\left(t\right)=\frac{value(t)-min}{max-min}$$

#### 4.3.3. Alert Generator

Alert generator is mainly responsible for triggering an alert in case a possible anomaly has been detected. Concretely, this system generates an alert when the new KPI sample has a noticeably different behavior compared to its normal behavior, and simultaneously, there is not a relevant behavioral change in the previous sample or in the sample from the previous period. Algorithm 1 presents the corresponding pseudocode.

Additionally, the alerts are classified into three types: low, medium and high. These levels depend on the severity of the behavioral change. They can be configured with the parameters introduced above: “th_low”, “th_med” and “th_high”. On the other hand, the system detects beneficial and harmful anomalies. Thus, an alert will be generated even if the KPI has been enhanced—e.g., if the download throughput per user has been increased.

**Algorithm 1:** Alert generator pseudocode.
1:
$d=\left|KPI^{\prime }\left(t\right)-avg\_buffer^{\prime }\left[mod\left(t,T\right)\right]\right|$
2:Storing “d” value at the end of “d_buffer”3:
**if** d>th_low **and** $(\left|d-d\_buffer[t-T]\right|>th\_low$ **or** $\left|d-d\_buffer[t-1]\right|>th\_low)$ **then**
4:     **if** d>th_high **then**5:        Triggering High Alert and Storing ’high’ at the end of “alert_buffer”6:     **else if** d>th_med **then**
7:        Triggering Medium Alert and Storing ’medium’ at the end of “alert_buffer”8:     **else**
9:        Triggering Low Alert and Storing ’low’ at the end of "alert_buffer"10:     **end if**
11:
**else**
12:     Storing ’no’ at the end of “alert_buffer” (no alert is triggered)13:
**end if**



#### 4.3.4. State Machine

**Figure 2:**
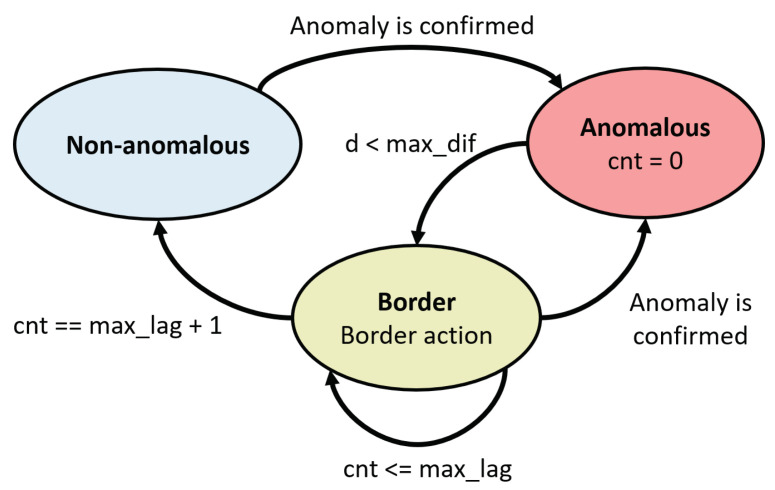
State machine.

Although State machine goes after Anomaly decision subsystem, it will be presented before for the sake of clarity. This stage allows one to track the state of a KPI, and therefore, to identify the end of an anomaly once its beginning has been indicated by Anomaly decision subsystem. Three states are defined:

- **Non-anomalous**: The current KPI behavior is similar to its normal average behavior. A KPI leaves this state in case Anomaly decision subsystem confirms the start of an anomaly.
- **Anomalous**: The current KPI behavior is anomalous. A KPI will leave this state as soon as a new incoming KPI sample is similar to its corresponding value stored in “avg_buffer”.
- **Border**: The KPI is leaving an anomalous period of time. If the KPI fulfills the conditions that can be seen in detail in Figure 2 and Algorithm 2, the KPI will definitively return to the non-anomalous state. These conditions monitor whether the new incoming KPI samples have actually returned to their normal values or whether the end of the anomalous state has been momentary. Otherwise, a KPI might come back to the anomalous state if Anomaly decision subsystem confirms a new anomaly.

**Algorithm 2:** Border action pseudocode.
1:
zero_samp=0
2:
**for** i=1,2,…,T **do**
3:     **if** $avg\_buffer^{\prime }\left[i\right]==0$ **then**
4:        zero_samp=zero_samp+1
5:     **end if**
6:
**end for**
7:
**if** d<max_dif **and** $(KPI^{\prime }\left(t\right)>0$ **or** zero_samp>T/2) **then**
8:     cnt=cnt+1
9:
**end if**



#### 4.3.5. Anomaly Decision Subsystem

This subsystem confirms whether there is an anomaly based on the generated alerts and the state of the KPI in the previous instants of time. The decision depends on three conditions detailed in Algorithm 3.

Line 1 indicates the first condition. It confirms an anomaly if a medium or high alert is triggered and other alert was generated in the previous “max_lag” samples. The second condition (line 3) confirms an anomaly if a low alert has been generated and another was triggered in the previous sample or a higher severity alert was generated in previous “max_lag” samples. Finally, line 5 indicates the last condition. It specifies that if the KPI state is “border”, and the KPI behavior is still different enough from the normal behavior, the KPI will return to the anomalous state. Therefore, it is necessary that two alerts have been generated to confirm an anomaly in the beginning. This will allow one to reduce the triggering of anomalies caused by sporadic outliers. In this way, the combined work of State machine and Anomaly decision subsystem will focus mainly on detecting and tracking the anomalies maintained over time in accordance with the network experts’ directives.

**Algorithm 3:** Anomaly decision subsystem pseudocode.
1:**if**(alert_buffer[t]==high**or**alert_buffer[t]==medium)**and** any alert in [t−max_lag,t−1] **then**2:     Anomaly is confirmed3:**else if**alert_buffer[t]==low**and**(alert_buffer[t−1]==low**or**alert_buffer[n]==high**or**alert_buffer[n]==medium for any n∈[t−max_lag,t−1]) **then**4:     Anomaly is confirmed5:
**else if** state_buffer[t]==border **and** d>th_med **then**
6:     Anomaly is confirmed7:
**else**
8:     No anomaly is confirmed9:
**end if**



#### 4.3.6. Normal Data Update

As explained before, the seasonal patterns may change or shift over time. Hence, a mechanism that automatically adapts the system to seasons of the year with different traffic patterns is required. Therefore, “avg_buffer” should be updated with each new incoming sample. However, since this buffer represents the non-anomalous values, it should be updated only with normal samples, disregarding those iterations in which the KPI state is “border” or “anomalous”. This update is carried out based on Equation (4). In this way, the proposed system will automatically adapt to long-term changes in the network without generating alerts.

(4) $$avg\_buffer_{updated}\left[mod\left(t,T\right)\right]=avg\_buffer\left[mod\left(t,T\right)\right]\cdot \left(1-\frac{T}{S}\right)+KPI\left(t\right)\cdot \frac{T}{S}$$

## 5. Evaluation Methodology

This section details the dataset and the performance metrics which are used to evaluate the feasibility of the proposed system. Given that the current use of commercial 5G networks is still quite low, tests have been carried out with data from a LTE Advanced network, without loss of generality. Furthermore, a last subsection further details the previously introduced open-source packages that are going to be compared with the proposed system.

### 5.1. Dataset

The present study has been carried out with a dataset of a real LTE Advanced mobile network. It covers 24,725 cells of a metropolitan area during 45 days. In this way, 650 performance measurements are collected for each cell with a fifteen minute time interval. These lower level information indicators have been used to calculate 240 KPIs with one hour granularity. It should be pointed out that if any sample of a performance measurement is not collected because of occasional technical errors, the KPI sample of the same hour will be calculated as the average of the rest of samples of the performance measurement collected in that hour. Following a network trouble, the engineers identified 80 cells with an unusually high number of anomalies in 15 key business related KPIs which were chosen in accordance with the network experts’ directives (Table 1). These cells had a mean of 12% anomalous samples, whereas mobile networks have a mean of about 3–4% anomalous samples in real-world scenarios [7]. In this way, 300 more cells have been labeled until the mean of anomalous samples from all labeled cells was consistent with this data. Hence, 15 KPIs of 380 labeled cells with a mean of 3.8% anomalous samples have been used to carry out the tests. As regards labeling, engineers have added a label to each hourly sample indicating whether or not it is anomalous. In this sense, they have identified different types of anomalies that are introduced as follows:

- **Outlier**: Occasional rare values that are usually collected for one or two hours straight. These can be severe spikes or drops.
- **Level shift anomaly**: The KPI mean suddenly increases or decreases over a long time interval, but the pattern of behavior is correlated with the usual one.
- **Ramp anomaly**: The KPI values are gradually changing over time. Hence, there are two different types of ramp anomalies. In the first type, the KPI behavior changes dramatically and then becomes more and more similar to normal behavior with each new sample. In contrast, the KPI behavior is normal but begins to change more and more with each new sample in the second type.
- **Pattern change**: The KPI behavior changes completely and the new behavior is not correlated with the normal one.

Therefore, long-term changes in the usual pattern of KPIs due to changes in user behavior during different months of the year are not considered anomalous. These changes are automatically addressed by Normal data update. Finally, it should be also noted that the dataset does not include synthetic anomalies. In contrast, the dataset does not attach any additional information about the reason for the anomalies—e.g., the cause of an anomalous increase in the number of connected users. Nonetheless, this type of information is included in some datasets. An IoT dataset is tagged with the cyber-attack corresponding to each anomaly in [21]. These anomalies can be compared with the anomalies introduced previously. For example, a large number of devices flood network resources to interrupt access to services in a denial of service (DoS) attack. This brute-force attack can be identified by the proposed method as a level shift anomaly in which the number of connected users suddenly shoots up.

### 5.2. Performance Metrics

The evaluation of the proposed system can be approached in two different ways. A system configuration can be proposed for each KPI or a global configuration for all KPIs. To demonstrate the feasibility of the proposed methodology, all KPIs will be used to achieve a single global configuration for all cells. With this purpose, the configuration will be based on different performance measures that can be calculated from the confusion matrix entries [22]. These entries are detailed as follows:

- **True positive (TP)**: Number of anomalous labeled samples which are correctly classified as anomalous by the system.
- **False positive (FP)**: Number of non-anomalous labeled samples which are wrongly classified as anomalous by the system.
- **True negative (TN)**: Number of non-anomalous labeled samples which are correctly classified by the system.
- **False negative (FN)**: Number of anomalous labeled samples which are wrongly classified by the system.

Hence, once the confusion matrix is obtained, five of the mostly cited performance measures in the machine learning literature can be calculated [15]. They are introduced below.

- **Accuracy**: Proportion of correctly classified samples of the total samples (Equation (5)).
- **Error rate**: Proportion of wrongly classified samples of the total samples (Equation (6)).
- **false Positive Rate (FPR)**: Proportion of non-anomalous labeled samples that have been wrongly classified (Equation (7)).
- **Precision**: Proportion of correctly classified anomalous samples of the total number of anomalous classified samples (Equation (8)).
- **Recall**: Proportion of correctly classified anomalous samples of all anomalous labeled samples (Equation (9)).

(5) $$Accuracy=\frac{TP+TN}{TP+TN+FP+FN}$$

(6) $$ErrorRate=\frac{FP+FN}{TP+TN+FP+FN}=1-Acc.$$

(7) $$FPR=\frac{FP}{TN+FP}$$

(8) $$Precision=\frac{TP}{TP+FP}$$

(9) $$Recall=\frac{TP}{TP+FN}$$

### 5.3. Other Analyzed Systems

To measure how good the performance of the proposed system is, it is compared with the performances of other systems previously among the state-of-the-art. These systems have the same goal as the proposed system, but they use different techniques. Given that they have been published as Python or R packages, the performances of all systems can be tested with the introduced dataset. Therefore, the pros and cons of each system will be exposed when tested in the following section, allowing us to draw more precise conclusions about the proposed system. These systems are described below:

- **Banpei**: This technique focuses on identifying new different behavior patterns and level shift anomalies maintained over time by using SST [16]. Thus, the detection of occasional outliers will be minimum.
- **ADTK**: This system uses different detection techniques to identify the maximum number of anomalies and a subsequent module combines the multiple lists of anomalies into the definitive one [17]. Hence, many anomalies may be detected by one or more techniques but not confirmed at the subsequent stage. The different techniques that the system uses are autoregression, detection based on percentiles, sliding window and principal component analysis (PCA).
- **Hochenbaum**: This technique uses S-H-ESD to detect any type of anomaly [18]. It calculates the statistical values that are used to identify anomalies based on the average percentage of anomalous samples that each KPI usually has. In this way, this system has been configured with the mean percentage of anomalous samples in the dataset, i.e., 3.8% of anomalous samples. However, the dataset used is unbalanced as indicated in Dataset subsection, so this system might not reach an optimal performance.

## 6. Results

In this section, the proposed system is evaluated with several tests. A configuration of the parameters of the online block is then proposed based on the results of the tests. Finally, a comparison between the proposed system with this settled configuration and the other introduced systems is addressed, and the final conclusions are drawn.

To that end, the 380 labeled cells have been divided into twenty sets of the same size to apply the k-fold cross-validation technique [23]. This technique aims at demonstrating the reliability and feasibility of the system with different training data in addition to maximizing the use of the available labeled data. Nevertheless, unlike most cases where this technique is applied, the least amount of data (one of twenty sets) was used to set up the configuration parameters of the online block to demonstrate that few labeled data are required for reliable performance. Hence, network operators would not have to spend a lot of resources on classifying data if they use the proposed method.

Before testing, the “S” value must be indicated based on the dataset size limitation. As the dataset contained 45 days in this study, 10 days was used in order to have a considerable percentage of data to calculate “avg_buffer” and a high amount of data for the tests. Therefore, 240 samples were used as the “S” value to calculate the normal behavior and to initialize the system.

**Figure 3:**
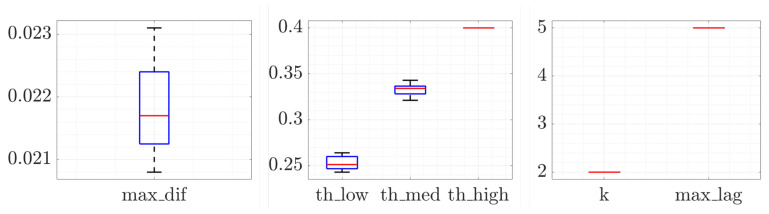
Optimal configuration of the parameters of the online block for each of the twenty training datasets of the k-fold cross-validation technique.

Since each of the twenty training datasets of the k-fold cross-validation technique was labeled, it was possible to calculate the optimal configuration of the parameters of the online block for each of these training datasets. Figure 3 shows all the optimal configurations that have been calculated for these twenty tests. It shows that these values are very similar even though the training data are not the same. Both “k” and “max_lag” values obtained have been the same for all the tests. On the other hand, it should be pointed out that “th_high” was manually configured once the rest of the configuration parameters have been calculated because it only decided the severity of the anomalies already confirmed. Once the optimal configuration of the parameters of each training dataset has been calculated, a single configuration of the parameters of the online block was proposed to be evaluated with the 380 labeled cells. This parameter configuration aimed at achieving a balanced performance of all the analyzed metrics. Therefore, the median values of the previous tests results have been proposed as configuration parameters.

**Figure 4:**
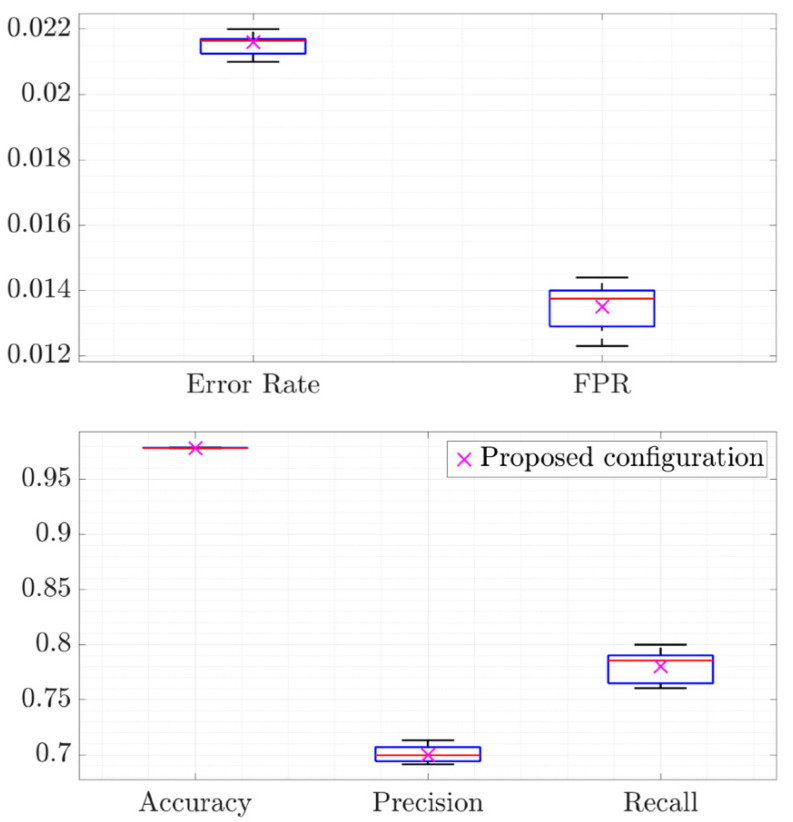
Performance of the proposed configuration and the rest of the configurations for the whole dataset.

Next, Figure 4 represents the system performance that the proposed configuration and the other configurations of the previous twenty tests achieved with the 380 labeled cells. The results indicate that the proposed system reaches a high level of accuracy, and therefore, a low error rate. Likewise, the recall is high at the same time as the FPR is low, which implies that most of the anomalies in the dataset are identified without shooting up the number of false alerts. Additionally, finally, even though the precision is less than the recall, most of the anomalies indicated by the proposed system are real. On the other hand, the variance is low for all the performance metrics except for the recall, which is slightly higher. This higher variance is given by the difference between a configuration that risks more or less at the time of identifying anomalies. In this sense, it is possible to modify the proposed configuration in order to decrease the FPR at the expense of decreasing the recall according to the network operator interests. Hence, if a less restrictive configuration is settled, a greater number of anomalies will be detected in exchange for increasing the FPR. Therefore, this new configuration could now detect the slight anomalies that had not been previously identified because the behavioral change was not severe enough to indicate an anomaly. On the other hand, the FPR would increase due to the detection of time shifts in user behavior and some occasional outliers that are not identified as anomalies by network experts. In addition, the tracking of KPIs may also indicate as anomalous some samples after an anomaly ends. For these reasons, a configuration such as the one proposed makes it possible to achieve a balanced overall performance compared with the performance obtained by the other configurations.

**Figure 5:**
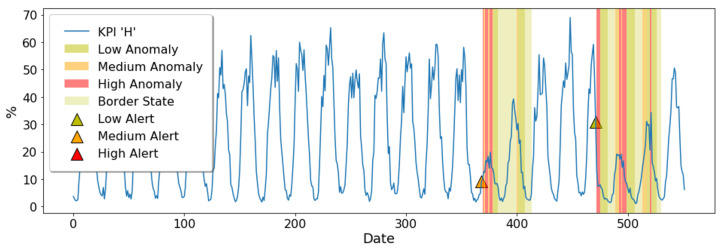
Example of visualization of the graphical user interface.

In addition, a graphical user interface has been proposed to monitor each KPI as can be seen in Figure 5. It shows the generated alerts and the KPI state. In this sense, the triangles represent the alerts triggered by Alert generator which have not been later confirmed as anomalies by Anomaly decision subsystem. On the contrary, if the samples are finally considered as anomalous, they are highlighted with colors, which represent the severity of the behavioral change in both the alerts and the anomalies. In a similar manner, a lighter green is also used in case the KPI state is “border”. Figure 5 shows an example of visualization where there are two level shift anomalies in the KPI H (Physical Resource Block usage in the downlink) that have been correctly identified by the proposed system. The resources usage is unusually low for two days at the first anomaly. On the other hand, the KPI drops unexpectedly during a central time of the day, and its values then remain too low for two more days at the second anomaly. Therefore, this interface would provide a user-friendly work environment for the network engineers to check that the anomalies have been correctly identified. In addition to the anomalies introduced above, network experts identify some anomalous spikes due to unusual overuse of network resources. However, the system does not detect them because the behavioral change is not large enough to be considered an anomaly due to the configuration used in this test. Hence, a less restrictive configuration could detect these anomalies; nevertheless, a greater number of FP might be reached. Thus, this example depicts that the current configuration achieves a trade-off between FP and FN.

**Figure 6:**
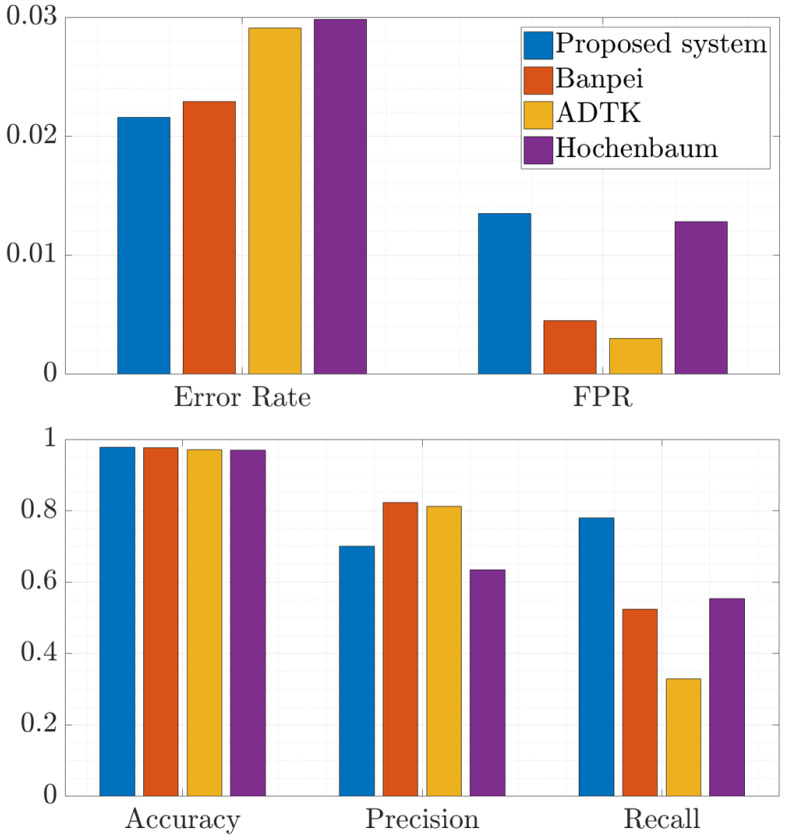
Comparison of the performance achieved by each approach.

Finally, Figure 6 shows a comparison of the performance of the proposed system and the rest of state-of-the-art systems with the same dataset. Firstly, it should be noticed that the accuracy—and therefore, the error rate—was similar for the proposed system and for Banpei because they are mainly focused on identifying level shift anomalies and anomalies with different behavioral patterns maintained over time, which are the most relevant in mobile networks. On the other hand, Hochenbaum achieved the worst performance in terms of these metrics because its use is improper for an unbalanced dataset. This is also reflected in FPR, since the number of FP will be high for KPIs that have a low level of anomalous samples. On the contrary, ADTK achieved the lowest FPR, since its requirement to decide whether a sample is anomalous or not is the hardest because of the combination of different anomaly detection techniques. This requirement was also noticed in the high precision of the method in the anomalies identified by the system. However, ADTK achieved the lowest recall, since many anomalies are not confirmed by the last stage of the system. Hence, most network anomalies are not identified by ADTK. Otherwise, the proposed system reached the highest FPR because the tracking of a KPI may imply that some samples are identified as anomalous after an anomaly finishes and the KPI returns to its normal state. In addition, the system may also indicate as anomalous some occasional outliers and slight time shifts in user behavior that are not considered as anomalies by network experts. Therefore, although the precision of the proposed system was not the highest due to the number of FP, it was the system that reached the highest recall. Hence, the proposed system identified the most network anomalies while maintaining a high level of precision, which is important to achieve the reduction of network OPEX. In this sense, the main difference between the proposed system performance and Banpei’s performance is that the latter only identifies the most severe anomalies maintained over time, so its FPR is lower and its precision is higher in exchange for a much lower recall. Finally, Hochenbaum does not stand out in terms of either precision or recall.

In summary, although Banpei and ADTK achieve low FPR and high precision, they do not identify enough anomalies. Regarding Hochenbaum, its performance is poor for this use case where some indicators may have many more anomalous samples than other indicators. Thus, sometimes it will detect most of the anomalies while triggering many FP, and other times it will identify few anomalies. Finally, even though the proposed system obtained a higher FPR and a slightly lower precision than other systems, it identified almost 80% of all network anomalies. This means that the overall performance of the proposed system is the best of the analyzed methods for use in mobile networks. Therefore, the proposed system allows the network operator to be aware of most of the anomalies, which implies that further optimization can be achieved in increasingly complex next-generation mobile networks.

## 7. Conclusions

In this paper, a system for detecting anomalies in mobile networks in real time has been presented. The system consists of several stages that allow one to distinguish and track harmful anomalies from occasional outliers. The system is focused mainly on the detection of new different behavior patterns and anomalies maintained over time according to network experts’ directives. In this sense, the system has been tested with a large dataset from a live LTE Advanced network where real anomalies have been labeled by network experts. Its performance has been then compared with the performances of other state-of-the-art anomaly detection systems.

Results have shown that the system can work feasibly without manual intervention once the configuration parameters have been set up based on a reduced set of labeled samples. The analyzed performance metrics show that the proposed methodology enables one to identify most network anomalies, while maintaining a high precision and a low level of false positives. In this sense, the proposed system takes a qualitative leap forward with respect to the rest of systems analyzed.

In addition, the proposed system might be deployed on existing base station equipment through a mobile network with low computing cost impact, which can lead to a reduction of network OPEX. In the same way, the proposed methodology can be extended to different radio access technologies. Likewise, it is possible to add a following phase that automatically uses this information to correlate anomalies of different KPIs in order to identify the root causes of the anomalies. The design of this block is left for future work.

## Figures and Tables

**Table 1:** Labeled key business related KPIs.

ID	Description
**A **	Average of Channel Quality Indicator
**B**	Average of connected users
**C**	Average of download throughput per user
**D**	Connection reestablishment attempts
**E**	Control Channel Element blocking rate
**F**	Control Channel Element usage rate
**G**	Download data traffic volume in Megabytes
**H**	Physical Resource Block usage in the downlink
**I**	Ping-pong handover rate
**J**	Reestablishment scheduling requests
**K**	Uplink data traffic volume in Megabytes
**L**	Uplink Received Signal Strength Indicator
	for the Physical Uplink Control Channel
**M**	Uplink Received Signal Strength Indicator
	for the Physical Uplink Shared Channel
**N**	VoLTE call setup success rate
**O**	VoLTE drop call rate

## Abbreviations

The following abbreviations are used in this manuscript:

ARIMA	Autoregressive Integrated Moving Average
DBSCAN	Density-based Spatial Clustering of Applications with Noise
DoS	Denial of Service
FN	False Negative
FP	False Positive
FPR	False Positive Rate
IoT	Internet of Things
KPI	Key Performance Indicator
NFV	Network Functions Virtualization
OPEX	Operating Expenses
PCA	Principal Component Analysis
SDN	Software Defined Networking
S-H-ESD	Seasonal Hybrid Extreme Studentized Deviate
SOM	Self-organizing Maps
SST	Singular Spectrum Transformation
TN	True Negative
TP	True Positive
